# Association Between Sexually Transmitted Infections and the Urine Culture

**DOI:** 10.5811/westjem.60033

**Published:** 2024-05-03

**Authors:** Johnathan M. Sheele, Carolyn Mead-Harvey, Nicole R. Hodgson

**Affiliations:** * Mayo Clinic, Department of Emergency Medicine, Jacksonville, Florida; † Mayo Clinic, Division of Clinical Trials and Biostatistics, Scottsdale, Arizona; ‡ Mayo Clinic Hospital, Department of Emergency Medicine, Phoenix, Arizona

**Keywords:** chlamydia, emergency department, emergency medicine, gonorrhea, *Trichomonas*, urinary tract infection

## Abstract

**Introduction:**

Bacterial urinary tract infections (UTI) and some sexually transmitted infections (STI) can have overlapping signs and symptoms or nonspecific findings, such as pyuria on urinalysis. Furthermore, results from the urine culture and the nucleic acid amplification test for an STI may not be available during the clinical encounter. We sought to determine whether gonorrhea, chlamydia, and trichomoniasis are associated with bacteriuria, information that might aid in the differentiation of STIs and UTIs.

**Methods:**

We used multinomial logistic regression to analyze 9,650 encounters of female patients who were aged ≥18 years and who underwent testing for STIs. The ED encounters took place from April 18, 2014–March 7, 2017. We used a multivariable regression analysis to account for patient demographics, urinalysis findings, vaginal wet-mount results, and positive or negative (or no) findings from the urine culture and testing for *Neisseria gonorrhoeae, Chlamydia trachomatis,* or *Trichomonas vaginalis.*

**Results:**

In multivariable analysis, infection with *T vaginalis*, *N gonorrhoeae,* or *C trachomatis* was not associated with having a urine culture yielding 10,000 or more colony-forming units per mililiter (CFU/mL) of bacteria compared with a urine culture yielding less than 10,000 CFU/mL or no urine culture obtained. The diagnosis of a UTI in the ED was not associated with having a urine culture yielding 10,000 or more CFU/mL compared with a urine culture yielding less than 10,000 CFU/mL.

**Conclusion:**

After adjusting for covariates, no association was observed between urine culture results and testing positive for trichomoniasis, gonorrhea, or chlamydia. Our results suggest that having a concurrent STI and bacterial UTI is unlikely.

Population Health Research Capsule What do we already know about this issue?

*There is an overlap in the signs, symptoms, and findings on urinalysis for women with urinary tract infections (UTIs) and sexually transmitted infections (STIs).*

What was the research question?

*For a woman suspected of having or found to have an STI, are the inflammatory changes observed on urinalysis most likely caused only by the STI, or could she have concurrent bacteriuria?*

What was the major finding of the study?

*After adjusting for covariates, no association was observed between urine culture results and testing positive for an STI, suggesting concurrent STI and bacterial UTI are unlikely.*

How does this improve population health?

*Concurrent STIs and bacterial UTIs are unlikely.*

## INTRODUCTION

Urinary tract infection (UTI) is one of the most common bacterial infections diagnosed in the emergency department (ED).^1^^,^^2^ Symptoms of UTI are the reason for approximately 1% of all ambulatory visits and result in 2–3 million ED visits in the US each year.^2^ Urine culture results can take more than a day, and the urinalysis findings can cause diagnostic uncertainty about the existence of a bacterial UTI. Adding to the problem is that the incidence of some sexually transmitted infections (STI) such as gonorrhea, chlamydia, and trichomoniasis is increasing in the US,^3^^,^^4^ and clinical manifestations of UTIs and STIs may overlap. These overlapping signs and symptoms may lead to underdiagnosing STIs in patients with urinary concerns and overtreating for STIs in patients with genital concerns.^5^^–^^9^ Previous study findings have shown that STIs are associated with sterile pyuria and other non-specific findings on urinalysis.^5^^,^^9^^,^^10^ Diagnostic confusion may be most common when trichomoniasis is identified in the ED by urinalysis or wet mount and the clinician must consider whether the urine inflammatory changes are caused by *Trichomonas vaginalis* only or by a concurrent bacterial UTI.

In this analysis, we sought to determine whether infection with gonorrhea, chlamydia, and trichomoniasis was associated with specific urine culture results. Specifically, we attempted to determine the frequency of STIs and having a urine culture yield 10,000 or more colony-forming units per milliliter (CFU)/mL) of bacteria. The research question we sought to answer was as follows: For a woman suspected of having or found to have gonorrhea, chlamydia, or trichomoniasis during the ED encounter who has genitourinary concerns, are the inflammatory changes observed on urinalysis most likely caused only by the STI, or is concurrent bacteriuria (eg, UTI) contributing?

## METHODS

### Dataset

We used an existing dataset of 75,000 ED encounters of patients ≥18 years in age from a single health system.^11^^–^^22^ All patients in the dataset received testing for gonorrhea, chlamydia, or trichomoniasis or underwent both urinalysis and urine culture. Patients undergoing only urinalysis, regardless of STI testing, were not included in the dataset. All ED encounters took place April 18, 2014–March 7, 2017. The dataset was created by the institution’s information technology team who extracted retrospective data from the electronic health records (EHR). For our analysis, we included women who were not admitted to the hospital and who had a nucleic acid amplification test (NAAT) for gonorrhea, chlamydia, or trichomoniasis or had a vaginal wet mount. Data on the NAAT swab site were not available. Individual patients could have more than one ED encounter. Our project was approved by the institutional review board with an exemption from full review, and informed consent was waived. Articles have been published using the original dataset.^11^^–^^22^

Patients in the dataset were identified as having trichomoniasis if the parasite was seen with urine microscopy (a method having very low sensitivity but high specificity), vaginal wet mount (moderate sensitivity and high specificity), or NAAT (high sensitivity and specificity).^22^^–^^24^ To avoid multicollinearity in the multivariable analysis, we consolidated findings from vaginal wet mount and urine microscopy for *T vaginalis* into a single variable labeled *T vaginalis* infection status known during the ED encounter. The *T vaginalis* NAAT (Aptima, Hologic, Inc, Marlborough, MA) result, or the *Neisseria gonorrhoeae* or *Chlamydia trachomatis* NAAT (Aptima), was considered separately because the result was not obtained until after the ED visit. Women may have tested positive for *T vaginalis* by more than one test during their encounter, and any patient with a positive *T vaginalis* test was considered to be infected with *T vaginalis.* All STI testing was performed at the discretion of the treating clinician.

We reported the vaginal wet mount as not performed if the patient had no results from the vaginal wet mount for white blood cells (WBC), yeast, *T vaginalis*, or clue cells. The vaginal wet mount WBCs were analyzed as 0–10/11 or more cells per high-power field (HPF).^16^ For the vaginal wet mount, yeast, clue cells, and *T vaginalis* were reported by the clinical laboratory as present or absent.

We considered a urinalysis to have been performed if any component test from the urinalysis was reported. The urine sample was reported to have been collected by the following methods: clean catch/voided; missing or not documented by nursing; or “other” (eg, bladder catheter, straight catheter, ileostomy, nephrostomy, suprapubic, or urostomy). From the urinalysis, we considered the following variables: bacteria (0–4+); blood (0–3+); glucose (positive or negative); ketones (positive or negative); leukocyte esterase level (0–3+); mucus (0–4+); nitrites (positive or negative); protein (positive or negative); red blood cells (RBC) (0–101 cells/HPF); *Trichomonas* (positive or negative); WBC clumps (positive vs negative); WBCs (0–101 cells/HPF); and yeast (present or absent). If a range of urine RBCs and WBCs was reported, we used the median of the range in the analysis, and if more than 100 cells/HPF were reported, we used the result “101 cells/HPF” for analysis. All urine tests were ordered at the discretion of the treating clinician.

We included the following demographic and triage information if it was available during the ED encounter: method of ED arrival; marital status; age; race; and the triage Emergency Severity Index. Age in years was converted to a categorical variable to account for the nonlinear relationship with STIs.^25^

Women were considered to have a UTI diagnosis if they had a specific ED code on the *International Classification of Diseases,* *9*^*th*^ *or 10*^*th*^ *Rev* *(ICD-9/ICD-10)* (Supplement 1). Women were considered pregnant if they had a documented positive pregnancy test or a specific *ICD-9* or *ICD-10* code (Supplement 1).

### Statistical Analysis

We summarized continuous variables as median and interquartile range, with analysis of variance *F* tests used to test associations. We reported categorical variables as counts and percentages, with a χ^2^ test used to test associations. We perfomed multinomial logistic regression analysis accounting for multiple demographic, clinical, and diagnostic testing variables, with the Wald test used to determine *P* values. Multivariable analyses were performed for patients who had complete data for all model covariates. Odds ratios and 95% confidence intervals were calculated from the multivariable model. A *P* value less than .05 was considered significant. We conducted statistical analyses with statistical software JMP Pro 14 (JMP Statistical Discovery, LLC, Cary, NC) and SAS version 9.4 (SAS Institute, Inc, Cary, NC).

## RESULTS

Among the 75,000 ED encounters in the original dataset, 16,755 women met our inclusion criteria. A summary of the clinical encounters is shown in Table 1. Among the 1,631 patient encounters with a positive test result for gonorrhea, chlamydia, or both, 1,443 (88.5%) had urinalysis, 443 (27.2%) had urine culture, and 438 (26.9%) had both urinalysis and urine culture. Among the 1,354 women with *T vaginalis* identified on vaginal wet mount and 418 women with a positive NAAT result for *T vaginalis*, 1,203 (88.8%) and 374 (89.5%) patients, respectively, had urinalysis. Table 2 shows encounters with a positive STI test result and the results of the urine culture. Among the 443 patients with gonorrhea, chlamydia, or both who had a urine culture result, 341 (77.0%) had less than 10,000 CFU/mL of bacteria, and 102 (23.0%) had 10,000 or more CFU/mL of bacteria in the urine culture.

**Table 1.** Demographics and clinical characteristics by urine culture result.

Characteristic	Total (N = 16,755)	No urine culture(n = 12,372)	Urine culture, <10,000 CFU/mL (n = 3,534)	Urine culture, ≥10,000 CFU/mL (n = 849)	P value
Age, y, no. (%)					.002^a^
18–28	10,524 (62.8)	7,769 (62.8)	2,201 (62.3)	554 (65.3)	
29–39	4,328 (25.8)	3,252 (26.3)	894 (25.3)	182 (21.4)	
≥40	1,903 (11.4)	1,351 (10.9)	439 (12.4)	113 (13.3)	
Race, no. (%)	(n = 16,683)	(n = 12,311)	(n = 3,523)		<.001^a^
Black	14,855 (89.0)	11,090 (90.1)	3,017 (85.6)	748 (88.1)	
Not Black	1,828 (11.0)	1,221 (9.9)	506 (14.4)	101 (11.9)	
Marital status, no. (%)	(n = 16,708)	(n = 12,336)	(n = 3,526)	(n = 846)	<.001^a^
Married or life partner	1,488 (8.9)	1,050 (8.5)	359 (10.2)	79 (9.3)	
Separated, divorced, or widowed	670 (4.0)	460 (3.7)	168 (4.8)	42 (5.0)	
Single	14,550 (87.1)	10,826 (87.8)	2,999 (85.1)	725 (85.7)	
Pregnant, no. (%)					<.001^a^
No	13,105 (78.2)	9,725 (78.6)	2,681 (75.9)	699 (82.3)	
Yes	3,650 (21.8)	2,647 (21.4)	853 (24.1)	150 (17.7)	
ESI, no. (%)	(n = 15,793)	(n = 11,810)	(n = 3,365)	(n = 798)	0.06^a^
1 and 2	353 (2.2)	255 (2.2)	77 (2.3)	21 (2.6)	
3	11,937 (74.7)	8,777 (74.3)	2,574 (76.5)	586 (73.4)	
4 and 5	3,683 (23.1)	2,778 (23.5)	714 (21.2)	191 (23.9)	
Mechanism of ED arrival, no. (%)	(n = 16,663)	(n = 12,309)	(n = 3,507)	(n = 847)	0.46^a^
EMS or police	1,122 (6.7)	815 (6.6)	244 (7.0)	63 (7.4)	
Public transportation or on foot	852 (5.1)	630 (5.1)	170 (4.8)	52 (6.1)	
Private vehicle	14,689 (88.2)	10,864 (88.3)	3,093 (88.2)	732 (86.4)	
Urine specimen source, no. (%)					<.001^a^
Clean catheter/voided urine	3,309 (19.7)	0 (0.0)	2,703 (76.5)	606 (71.4)	
Other	71 (0.4)	0 (0.0)	51 (1.4)	20 (2.4)	
Not documented or missing	13,375 (79.8)	12,372 (100.0)	780 (22.1)	223 (26.3)	
NAAT for *Chlamydia trachomatis,* no. (%)					<.001^a^
Negative	14,985 (89.4)	11,123 (89.9)	3,127 (88.5)	735 (86.6)	
Positive	1,303 (7.8)	958 (7.7)	266 (7.5)	79 (9.3)	
No test result	467 (2.8)	291 (2.4)	141 (4.0)	35 (4.1)	
NAAT for *Neisseria gonorrhoeae*, no. (%)					<.001^a^
Negative	15,819 (94.4)	11,745 (94.9)	3,292 (93.2)	782 (92.1)	
Positive	477 (2.8)	342 (2.8)	104 (2.9)	31 (3.7)	
No test result	459 (2.7)	285 (2.3)	138 (3.9)	36 (4.2)	
NAAT for *Trichomonas vaginalis,* no. (%)					<.001^a^
Negative	4,505 (26.9)	3,409 (27.6)	854 (24.2)	242 (28.5)	
Positive	418 (2.5)	293 (2.4)	94 (2.7)	31 (3.7)	
No test result	11,832 (70.6)	8,670 (70.1)	2,586 (73.2)	576 (67.8)	
Diagnosed with UTI in the ED, no. (%)					<.001^a^
No	14,849 (88.6)	11,456 (92.6)	2,900 (82.1)	493 (58.1)	
Yes	1,906 (11.4)	916 (7.4)	634 (17.9)	356 (41.9)	
Treatment of gonorrhea and chlamydia, no. (%)					0.82^a^
No	13,593 (81.1)	10,051 (81.2)	2,855 (80.8)	687 (80.9)	
Yes	3,162 (18.9)	2,321 (18.8)	679 (19.2)	162 (19.1)	
Vaginal wet mount, WBCs/HPF, no. (%)					<.001^a^
11–100	5,296 (31.6)	3,716 (30.0)	1,287 (36.4)	293 (34.5)	
≤10	10,868 (64.9)	8,233 (66.5)	2,119 (60.0)	516 (60.8)	
Not performed	591 (3.5)	423 (3.4)	128 (3.6)	40 (4.7)	
Vaginal wet mount, yeast, no. (%)					0.21^a^
Present	1,027 (6.1)	762 (6.2)	217 (6.1)	48 (5.7)	
None	14,538 (86.8)	10,765 (87.0)	3,036 (85.9)	737 (86.8)	
Not performed	1,190 (7.1)	845 (6.8)	281 (8.0)	64 (7.5)	
Vaginal wet mount, clue cells, no. (%)					<.001^a^
None	8,826 (52.7)	6,449 (52.1)	1,908 (54.0)	469 (55.2)	
Present	6,941 (41.4)	5,232 (42.3)	1,386 (39.2)	323 (38.0)	
Not performed	988 (5.9)	691 (5.6)	240 (6.8)	57 (6.7)	
Leukocyte esterase (urine)					<.001^b^
No. (missing)	14,616 (2,139)	10,381 (1,991)	3,403 (131)	832 (17)	
Median (IQR)	0.0 (0.0–1.0)	0.0 (0.0–1.0)	1.0 (0.0–2.0)	2.0 (1.0–3.0)	
Range	0.0–3.0	0.0–3.0	0.0–3.0	0.0–3.0	
Nitrite (urine), no. (%)	(n = 14,818)	(n = 10,505)	(n = 3,480)	(n = 843)	<.001^a^
Negative	14,257 (96.2)	10,236 (97.4)	3,417 (98.5)	604 (71.6)	
Positive	561 (3.8)	269 (2.6)	53 (1.5)	239 (28.4)	
WBCs (urine)					<.001^b^
No. (missing)	10,692 (6,063)	7,199 (5,173)	2,699 (835)	794 (55)	
Median (IQR)	5.0 (2.5–13.0)	3.0 (2.5–12.5)	8.0 (2.5–16.0)	31.5 (10.0–101.0)	
Range	0.0–101.0	0.0–101.0	0.0–101.0	0.0–101.0	
Bacteria (urine)					<.001^b^
No. (missing)	10,688 (6,067)	7,194 (5,178)	2,700 (834)	794 (55)	
Median (IQR)	1.0 (0.0–1.0)	1.0 (0.0–1.0)	1.0 (0.0–1.0)	1.0 (1.0–2.0)	
Range	0.0–4.0	0.0–4.0	0.0–4.0	0.0–4.0	
Blood (urine)					<.001^b^
No. (missing)	14,604 (2,151)	10,361 (2,011)	3,411 (123)	832 (17)	
Median (IQR)	0.0 (0.0–1.0)	0.0 (0.0–1.0)	0.0 (0.0–1.0)	1.0 (0.0–2.0)	
Range	0.0–3.0	0.0–3.0	0.0–3.0	0.0–3.0	
Glucose (urine), no. (%)	(n = 14,809)	(n = 10,500)	(n = 3,467)	(n = 842)	0.39^a^
Negative	14,216 (96.0)	10,092 (96.1)	3,322 (95.8)	802 (95.2)	
Positive	593 (4.0)	408 (3.9)	145 (4.2)	40 (4.8)	
Ketones (urine), no. (%)	(n = 14,786)	(n = 10,477)	(n = 3,467)	(n = 842)	<.001^a^
Negative	12,220 (82.6)	8,740 (83.4)	2,808 (81.0)	672 (79.8)	
Positive	2,566 (17.4)	1,737 (16.6)	659 (19.0)	170 (20.2)	
Mucus (urine)					0.88^b^
No. (missing)	10,692 (6,063)	7,202 (5,170)	2,696 (838)	794 (55)	
Median (IQR)	1.0 (0.0–2.0)	1.0 (0.0–2.0)	1.0 (0.0–2.0)	1.0 (0.0–2.0)	
Range	0.0–4.0	0.0–4.0	0.0–4.0	0.0–4.0	
Protein (urine), no. (%)	(n = 14,800)	(n = 10,494)	(n = 3,464)	(n = 842)	<.001^a^
Negative	10,553 (71.3)	7,716 (73.5)	2,366 (68.3)	471 (55.9)	
Positive	4,247 (28.7)	2,778 (26.5)	1,098 (31.7)	371 (44.1)	
RBCs (urine)					<.001^b^
No. (missing)	10,693 (6,062)	7,196 (5,176)	2,701 (833)	796 (53)	
Median (IQR)	2.5 (2.0–12.5)	2.5 (1.0–12.5)	2.5 (2.0–12.5)	5.0 (2.3–22.8)	
Range	0.0–101.0	0.0–101.0	0.0–101.0	0.0–101.0	
WBC clumps (urine), no. (%)	(n = 10,578)	(n = 7,116)	(n = 2,672)	(n = 790)	<.001^a^
None	10,118 (95.7)	6,915 (97.2)	2,549 (95.4)	654 (82.8)	
Present	460 (4.3)	201 (2.8)	123 (4.6)	136 (17.2)	
Yeast (urine), no. (%)	(n = 10,628)	(n = 7,154)	(n = 2,684)	(n = 790)	0.04^a^
Present	280 (2.6)	171 (2.4)	80 (3.0)	29 (3.7)	
None	10,348 (97.4)	6,983 (97.6)	2,604 (97.0)	761 (96.3)	
*T vaginalis* status during ED encounter, no. (%)	(n = 16,308)	(n = 11,987)	(n = 3,478)	(n = 843)	<.001^a^
No wet mount performed	720 (4.4)	451 (3.8)	216 (6.2)	53 (6.3)	
Negative^c^	14,176 (86.9)	10,554 (88.0)	2,922 (84.0)	700 (83.0)	
Positive	1,412 (8.7)	982 (8.2)	340 (9.8)	90 (10.7)	

^a^ χ^2^ test.

^b^ Analysis of variance *F* test.

^c^ Negative vaginal wet mount and urine microscopy (if performed).

*CFU*, colony-forming units; *ED*, emergency department; *EMS*, emergency medical services; *ESI*, Emergency Severity Index; *HPF*, high-power field; *NAAT*, nucleic acid amplification test; *RBCs*, red blood cells; *UTI*, urinary tract infection; *WBCs*, white blood cells.

**Table 2.** Positive STI test results by urine culture.^a^

		Positive for *Trichomonas vaginalis* by test method			
Urine culture result, CFU/mL	Positive for gonorrhea, chlamydia, or both on NAAT (n = 1,631)	Urine microscopy (n = 275)	Vaginal wet mount (n = 1,354)	NAAT (n = 418)	
No urine culture	1,188 (72.8)	186 (67.6)	943 (69.6)	293 (70.1)
0−<10,000	341 (20.9)	71 (25.8)	326 (24.1)	94 (22.5)
10,000−<100,000	15 (0.9)	5 (1.8)	16 (1.2)	6 (1.4)
>100,000	87 (5.3)	13 (4.7)	69 (5.1)	25 (6.0)

^a^ Data is presented as No. (%). Women may have tested positive for *T vaginalis* by more than 1 test.

*CFU*, colony-forming units; *NAAT*, nucleic acid amplification test; *STI*, sexually transmitted infection.

In total, 1,804 patient encounters had a positive test result for *Trichomonas* by urine microscopy, vaginal wet mount, or NAAT. Of these, 1,612 (89.4%) had a urinalysis test result, 548 (30.4%) had a urine culture performed, and 538 (29.8%) had both a urinalysis and urine culture result. A total of 9,650 clinical encounters had complete observations for all model covariates and were included in the multivariable analysis (Table 3). This number included 2,414 patient encounters with less than 10,000 CFU/mL of bacteria, 722 patients with 10,000 or more CFU/mL of bacteria, and 6,514 patients with no urine culture performed.

**Table 3.** Multinomial logistic regression using urine culture result as the outcome variable (N = 9,650).

Variable	Comparison group	Reference	≥10,000 CFU/mL vs <10,000 CFU/mL		≥10,000 CFU/mL vs no urine culture done	
			OR (95% CI)	*P* value^a^	OR (95% CI)	*P* value^a^
Age, y^b^	29–39	18–28	0.82 (0.65–1.04)	0.10	0.83 (0.67–1.04)	0.11
	≥40	18–28	0.86 (0.63–1.17)	0.33	0.99 (0.74–1.34)	0.97
Marital status	Married or life partner	Single	1.32 (0.95–1.83)	0.10	1.43 (1.05–1.95)	0.02
	Separated, divorced, or widowed	Single	1.04 (0.66–1.64)	0.87	1.28 (0.83–1.99)	0.26
Pregnant	Yes	No	0.80 (0.62–1.02)	0.08	1.02 (0.80–1.29)	0.89
ESI	3	1 and 2	1.42 (0.74–2.72)	0.30	1.32 (0.71–2.47)	0.38
	4 and 5	1 and 2	1.42 (0.72–2.80)	0.31	1.30 (0.68–2.48)	0.43
NAAT for *Chlamydia trachomatis*	Positive	Negative	0.97 (0.70–1.35)	0.86	0.87 (0.64–1.18)	0.37
	No test result	Negative	0.87 (0.17–4.55)	0.87	1.27 (0.26–6.35)	0.77
NAAT for *Neisseria gonorrhoeae*	Positive	Negative	0.86 (0.52–1.42)	0.56	0.86 (0.53–1.37)	0.52
	No test result	Negative	1.20 (0.23–6.31)	0.83	1.23 (0.25–6.13)	0.80
NAAT for *Trichomonas vaginalis*	Positive	Negative	0.90 (0.53–1.52)	0.69	0.95 (0.58–1.55)	0.83
	No test result	Negative	0.73 (0.59–0.90)	.004	0.77 (0.63–0.94)	0.01
Race	Black	Non-Black	1.27 (0.95–1.70)	0.10	0.92 (0.70–1.22)	0.56
RBCs (urine)	1-Unit increase		1.00 (0.99–1.00)	0.40	1.00 (1.00–1.00)	0.66
Mechanism of ED arrival	EMS/police	Private vehicle	0.90 (0.62–1.29)	0.56	1.00 (0.71–1.42)	0.99
	Public transportation/on foot	Private vehicle	1.06 (0.71–1.58)	0.77	1.17 (0.80–1.71)	0.41
Diagnosed with UTI in the ED	Yes	No	1.17 (0.94–1.45)	0.16	2.05 (1.68–2.51)	<.001
Treatment of gonorrhea and chlamydia	Yes	No	0.79 (0.62–1.02)	0.07	0.85 (0.67–1.07)	0.17
*T vaginalis* status during ED encounter	No wet mount performed	Negative^c^	0.77 (0.17–3.53)	0.73	0.50 (0.12–2.14)	0.35
	Positive	Negative^c^	0.87 (0.63–1.20)	0.41	0.76 (0.56–1.04)	0.08
Bacteria (urine)	1-Unit increase	None	1.13 (1.05–1.23)	<.001	1.19 (1.10–1.28)	<.001
Blood (urine)	1-Unit increase	None	1.15 (1.04–1.27)	.006	1.03 (0.94–1.13)	0.58
Glucose (urine)	Positive	Negative	0.91 (0.59–1.42)	0.68	0.98 (0.65–1.49)	0.93
Ketones (urine)	Positive	Negative	1.03 (0.80–1.31)	0.83	1.13 (0.90–1.42)	0.31
Leukocyte esterase (urine)	1-Unit increase	None	0.98 (0.88–1.09)	0.66	1.16 (1.05–1.29)	<.001
Mucus (urine)	1-Unit increase	None	0.99 (0.92–1.06)	0.81	0.98 (0.92–1.05)	0.62
Nitrite (urine)	Positive	Negative	15.7 (10.8–22.76)	<.001	5.72 (4.45–7.34)	<.001
Protein (urine)	Positive	Negative	0.71 (0.57–0.88)	.002	0.76 (0.62–0.93)	<.001
WBC clumps (urine)	Present	None	1.54 (1.10–2.15)	0.01	1.89 (1.39–2.56)	<.001
Yeast (urine)	Present	None	1.18 (0.70–1.98)	0.53	1.33 (0.82–2.18)	0.25
WBCs (urine)	1-Unit increase	None	1.02 (1.02–1.02)	<.001	1.02 (1.02–1.02)	<.001
Vaginal wet mount, clue cells	Present	None	0.91 (0.75–1.11)	0.35	0.78 (0.65–0.94)	<.001
	Not performed	None	0.33 (0.06–1.70)	0.19	0.53 (0.12–2.43)	0.42
Vaginal wet mount, WBCs/HPF	11–100	≤10	0.68 (0.55–0.84)	<.001	0.84 (0.69–1.02)	0.09
	Not performed	≤10	1.01 (0.36–2.88)	0.98	0.96 (0.35–2.64)	0.94
Vaginal wet mount, yeast	Present	None	0.96 (0.64–1.42)	0.82	0.77 (0.53–1.12)	0.17
	Not performed	None	2.68 (0.50–14.45)	0.25	3.50 (0.74–16.59)	0.11

^a^ Covariate Wald test from the multinomial logistic regression model.

^b^ Age was grouped as 18–28, 29–39, and ≥40 years.

^c^ Negative test result by vaginal wet mount and urine microscopy (if performed).

*CFU*, colony-forming units; *ED*, emergency department; *EMS*, emergency medical services; *ESI*, Emergency Severity Index; *HPF*, high-power field; *NAAT*, nucleic acid amplification test; *OR*, odds ratio; *RBCs*, red blood cells; *UTI*, urinary tract infection; *WBC*, white blood cell.

The following variables were significantly more likely to be associated with a urine culture with 10,000 or more CFU/mL compared with less than 10,000 CFU/mL: higher bacteriuria on urinalysis; higher amount of blood in the urine nitrite-positive urine presence of urinary WBC clumps; higher urinary WBC count; and fewer WBCs on the vaginal wet mount (all *P* ≤ 0.01; Table 3). These variables had a significantly lower likelihood of being associated with a urine culture with ≥10,000 CFU/mL: no *T vaginalis* NAAT result (compared with a negative *T vaginalis* NAAT) and protein in the urine (both *P* ≤ 0.01; Table 3). The following variables were significantly more likely to be associated with ≥10,000 CFU/mL of bacteria in the urine culture (compared with no urine culture performed): married/life partner (vs single); higher bacteriuria on urinalysis; higher urine leukocyte esterase level; nitrite-positive urine, protein in the urine, presence of urinary WBC clumps, UTI diagnosed in the ED, and higher urinary WBC count (all *P* ≤ 0.02; Table 3). These variables had a significantly lower likelihood of being associated with a urine culture with ≥10,000 CFU/mL (compared with no urine culture performed): no *T vaginalis* NAAT result (compared with a negative *T vaginalis* NAAT), protein in the urine, and no vaginal wet mount clue cells (compared with present) (all *P* ≤ 0.01; Table 3). *Neisseria gonorrhoeae* or *C trachomatis detected by NAAT,* or known *T vaginalis* infection in the ED, was not associated with a urine culture yielding 10,000 or more CFU/mL. Additionally, UTI diagnosed in the ED was not associated with a urine culture yielding 10,000 or more CFU/mL compared with less than 10,000 CFU/mL.

## DISCUSSION

Both UTIs and STIs can have overlapping signs and symptoms and can cause inflammatory changes in the urine. Distinguishing between UTI and STI can be challenging in the ED.^5^^,^^6^^,^^26^ We sought to assess the relationship between bacteriuria and STIs. Our research question was as follows: For a woman suspected of having or found to have gonorrhea, chlamydia, or trichomoniasis during the ED encounter who has genitourinary concerns, are the inflammatory changes observed on urinalysis most likely caused only by the STI, or is concurrent bacteriuria (eg, UTI) contributing? Our results show that infection with gonorrhea, chlamydia, or trichomoniasis was not associated with also having a urine culture yielding ≥10,000 CFU/mL of bacteria compared with <10,000 CFU/mL or no urine culture performed. An important finding was that when *T vaginalis* was identified during the ED encounter on urine microscopy or vaginal wet mount, there was no significant association with bacteria in the urine culture. When an emergency clinician is evaluating a woman with genitourinary concerns and an STI is suspected or actually identified, as is the case on urine microscopy or vaginal wet mount for *T vaginalis,* bacteriuria is not more likely to coexist. Our findings support recommendations for screening for both UTIs and STIs in appropriate patients.^7^^,^^8^ For instance, women undergoing pelvic examination who were also diagnosed with a UTI in the ED were subsequently found to have high rates of STIs.^7^ However, emergency clinicians frequently do not screen for STIs in women with dysuria who are diagnosed with a UTI.^8^ Furthermore, our findings support those of smaller studies.^6^^,^^27^

A study by Prentiss et al showed that among adolescent girls with urinary tract symptoms in the ED, 9% had an STI, 57% had a UTI, and 6% had both an STI and a UTI.^6^ Clinician accuracy was 83% for STIs and 71% for UTIs, whereas only 23% correctly diagnosed patients with both UTI and STI.^6^ Shapiro et al^27^ found that among 92 women with urinary tract symptoms, STI rates were not different between women with a positive vs a negative urine culture (10^2^ CFU/mL). Additionally, a retrospective study of ED patients found that patients who were treated for a UTI, tested positive for gonorrhea, chlamydia, or trichomoniasis, and had pyuria were significantly more likely to have a negative urine culture than a positive urine culture.^9^ Reliance on positive urine nitrite and pyuria to treat for UTI in patients with confirmed or suspected STI may result in overtreatment with antibiotics. However, patients with an STI and a positive urine culture had significantly higher urine leukocytes than those with negative culture results.^9^

We also found that clinical encounters in which patients were diagnosed with a UTI in the ED were not more likely to have a urine culture of ≥10,000 CFU/mL of bacteria compared with <10,000 CFU/mL Possibly, patients who were diagnosed with a UTI but who had <10,000 CFU/mL were more likely to have an STI, but this association was not examined in the current study. Because the diagnosis of a UTI was not part of our inclusion criteria, not all women with a UTI diagnosis are represented in our cohort. We were able to study only women who had both a urinalysis and urine culture, not just a urinalysis. Therefore, the association between a UTI diagnosis and bacteriuria deserves further investigation.

## LIMITATIONS

Although our study used a large dataset, it has some limitations. First, not all women from our dataset underwent urinalysis, urine culture, vaginal wet mount, and NAAT for STIs. Furthermore, not all women diagnosed with a UTI underwent STI testing or a vaginal wet mount. Second, modeling *T vaginalis* in the ED has inherent limitations because the urinalysis and vaginal wet-mount results are available to the clinician during the encounter, but they lack high sensitivity, whereas NAAT is highly sensitive and specific but typically does not yield results during the patient encounter. Third, women undergoing STI testing who also had a urine culture may have been more likely to be concerned about urinary symptoms, which could have biased our analysis to those women with genitourinary concerns. Because we were unable to include history and physical examination findings in our analysis, we could not differentiate between patients with more genital concerns and those having more urinary symptoms. Alternatively, some women included in the analysis may have had asymptomatic bacteriuria or an asymptomatic STI, although this possibility is thought to be less likely. We did not attempt to differentiate between contaminated urine cultures and those yielding classic uropathogens.^5^

Fourth, our dataset represented a limited geographical area in the US, and the patients were predominantly Black; therefore, our results may not be generalizable to other locations and races. Fifth, the data did not include pediatric patients or men; so our results cannot be extrapolated to those groups. Sixth, patients who were treated presumptively for STIs without specific testing were excluded from analysis, and this could have resulted in selection bias. Finally, inherent limitations exist to using a dataset extracted from the institution’s EHR and *ICD* codes.

## CONCLUSION

In our regression analysis, positive gonorrhea, chlamydia, and trichomoniasis test results were not associated with bacteriuria yielding ≥10,000 CFU/mL compared with <10,000 CFU/mL or no urine culture obtained.

## Supplementary Information




