# Supplementary material for: Association Between Sexually Transmitted Infections and the Urine Culture
Source: West J Emerg Med. 2024 May 3;25(3):358–67. doi: 10.5811/westjem.60033 (PMC11112662; doi:10.5811/westjem.60033)
Supplement: Supplementary file 1 [file wjem-25-358-s001.docx]

Supplement 1.

UTI related ICD codes: N30.90, O86.22, N30.00, N30.91, N30, N30.0, N30.01, N30.9, O23.10, O86.20, N39.0, O08.83, O03.38, O04.88, O03.88, O86.2, O86.29, O23.40, 646.64, 599.0, 639.8, 646.60, 595.0, 595.9, 595.89, or 595.0.

Pregnancy related ICD codes: O00.1, O00.8, O21.9, O00.90, Z32.01, O00, O20, 643.93, 633.90, V72.42.
